# Future Horizons for Neurodevelopmental Disorders: Placental Mechanisms

**DOI:** 10.3389/fped.2021.653230

**Published:** 2021-04-08

**Authors:** Sreelekha Kundu, Sara V. Maurer, Hanna E. Stevens

**Affiliations:** Department of Psychiatry, University of Iowa Carver College of Medicine, Iowa City, IA, United States

**Keywords:** placenta, neurodevelopmental disorders, pregnancy, brain development, prenatal programming

## Introduction

To improve care for the estimated 17.1 million children with psychiatric disorders in the United States (1), it is critical to explore all possible connections to better understand these disorders' origins. The onset of neurodevelopmental/psychiatric disorders varies person-to-person. However, even for disorders diagnosed after infancy, there is a growing appreciation that the origins of these disorders are at the earliest stages of brain development—prenatally. Furthermore, not only is it crucial to understand *what* is unusual during development, but also *why* this occurs.

**Figure 1:**
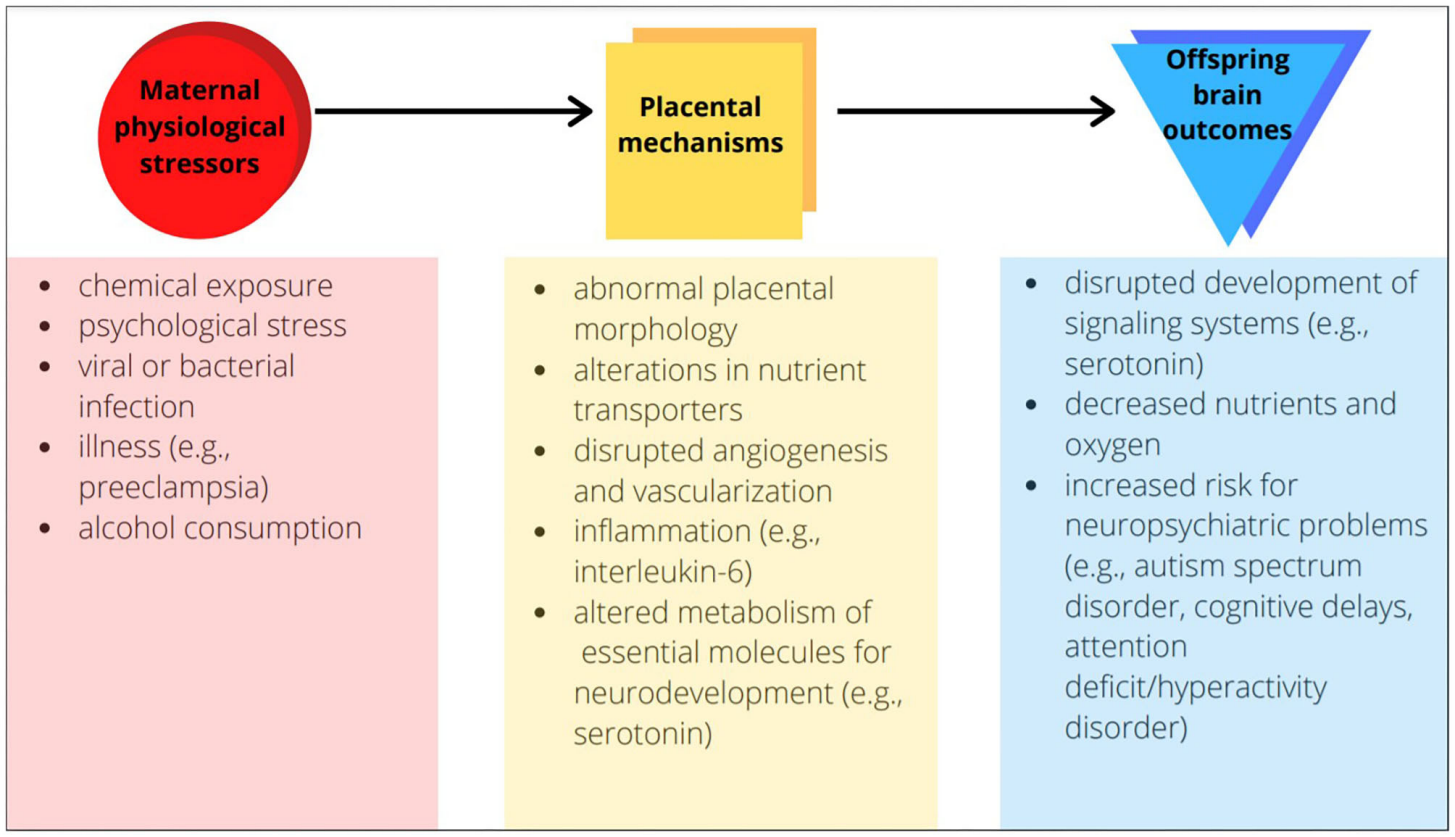
Examples of placental mechanisms as the link between maternal physiological stressors and offspring brain outcomes.

A significant contributor to abnormal prenatal brain development is physiological stress during pregnancy (2–6). Physiological stress induces a significant shift from homeostasis, and may arise from chemical exposures, psychological stress, infections, and illnesses such as preeclampsia or gestational diabetes. Epidemiological studies link maternal stress with offspring neurodevelopmental impairments (2), and animal studies have demonstrated causality of this relationship. For example, preeclampsia—a disorder with disrupted maternal vascular and immune biology—increases risk of neuropsychiatric problems among children (3). Evidence has come from human and non-human preeclampsia studies implicating *what* in the offspring brain has changed: its morphology, white matter, and vasculature. When we ask the further question of *why* these changes occur with preeclampsia or any maternal stress, it is critical to consider the biology of not only mother and offspring but also their link—the placenta. Changes in placenta may be a critical factor for offspring neurodevelopment (Figure 1).

The placenta forms after conception and is delivered along with the offspring. During gestation, placenta serves as the mediator between mother and fetus, supplying nutrients and oxygen to the fetus and removing waste and CO_2_. The role of the placenta has been emphasized previously, and continues to warrant attention when examining disorders with developmental origins (7). Many cultures bury the placenta after birth, out of respect for its role as a “guide” through pregnancy or its link to the child's future (8). The level of respect for the placenta these cultures offer seems fitting, as growing evidence suggests its importance in long-term neurodevelopmental outcomes.

## Placental Biology

The nutrient and waste exchange of the placenta that supports fetal development is just part of its critical role. The placenta also produces critical hormones, growth factors, proteins for metabolizing endogenous molecules (e.g., 11βHSD2 breakdown of elevated cortisol in normal pregnancy to limit fetal exposure) or transporting exogenous factors (e.g., efflux transporters for xenobiotic chemicals), and other molecular substrates (e.g., such as serotonin which is directly supplied to the fetal brain (9–11). All of these factors impact fetal development and regulate communication between the separate but linked biological domains of the mother and fetus. These processes of normal pregnancy are dependent on proper placental structural formation and its function throughout gestation (12). Maternal physiological factors may influence the structure and function of the placenta's unique connection between mother and baby. To better understand the placenta, it is important to understand general periods of placental development:

Beginning of Gestation: Placental villi are multi-layered folds of tissue in which fetal and maternal blood vessels come in close contact (13). Villous and extravillous structures form as a result of the proliferation and differentiation of trophoblast cells, which arise during the earliest divisions of cells in the embryo. The villous and extravillous structures physically anchor the placenta into the uterus and allow gas and nutrient exchange and other functions.

First Half of Gestation: Trophoblasts undergo the most changes. For example, some trophoblasts replace endothelial cells that make up the uterine spiral arteries to ensure adequate fetal blood supply. This mechanism also serves to protect the placenta from potentially harmful fluctuations in oxygen levels (14).

Second Half of Gestation: Extensive angiogenesis and vascularization occurs. The formation of new blood vessels allows blood to enter the trophoblast cell-lined sinuses in the uterus and continue to meet the nutritional and other physiological needs of the growing offspring (15).

## Gestational Diabetes

Abnormal placental morphology and function have significant impacts on fetal outcome. For example, gestational diabetes mellitus (GDM) has been linked to offspring risk for metabolic, cardiovascular, and neuropsychiatric problems including autism spectrum disorder (ASD), ADHD, depression, anxiety, and cognitive delay (16). Maternal diabetic abnormalities may have direct impacts on fetal metabolism, changing levels of specific nutrients and hormones. However, studies have also found in GDM models that maternal hyperglycemia leads to decreased placental glucose transporters (17). This in turn may lead to decreased fetal glucose, further leading to delays, as glucose is a critical nutrient (13). GDM also induces altered metabolism and placental development and function at early stages which may be responsible for excessive fetal growth (13). Specifically, inflammatory and cellular stress pathways in placental cells such as NF-κB signaling or ER stress likely play a role in GDM (18). Evidence suggests that abnormal maternal metabolism stimulates both adipose and placental cells, increasing production of inflammatory cytokines that then may influence the fetus in multiple ways (19).

## Maternal Inflammation

Other maternal factors, including bacterial or viral infections such as influenza, can elicit increased inflammatory cytokines which may alter placental function. Maternal immune activation (MIA) during pregnancy has been linked with an increased risk for neuropsychiatric risk in offspring, including ASD (20).

Animal models show that influenza infection during pregnancy alters the placenta in multiple ways. After maternal influenza, overall placental growth is reduced (21), as well as reduced labyrinth zone thickness, suggesting that disrupted vascular development of the placenta occurs and then likely reduces nutrient and oxygen exchange (22). Maternal influenza also alters the expression of a significant number of placental genes, mainly implicating inflammatory, immune, and hypoxia pathways but also overlapping with risk genes for neuropsychiatric disorders (23). In these studies, placentas also showed cellular abnormalities including thrombi and elevated immune cell number. Offspring brain showed persistent changes in some key neuronal genes many weeks after this maternal exposure (23). At the same time, no viral genes were found in placenta or offspring brain, suggesting indirect pathways for brain alterations such as placental morphological and functional changes.

## Fetal Alcohol Spectrum Disorder

Fetal Alcohol Spectrum Disorder (FASD) affects up to 1.5 of every 1,000 births in the United States (24). FASD includes low body weight, poor coordination, and cardiovascular complications. Children with FASD experience neurodevelopmental challenges such as hyperactive behavior, poor memory, and speech and language delays. As with other maternal physiological disruptions, the role of the placenta may be critical for impacts of gestational alcohol exposure.

Disruptions to placental vascular structure and function may be a mechanism involved in the origins of FASD. Increased glucocorticoid levels with alcohol consumption (25) may play a role in reduced blood flow, given known impacts of cortisol on placental angiogenesis (26). In human placentae from pregnancies in which women were prospectively assessed to have used alcohol, levels of two angiogenic proteins vascular endothelial growth factor receptor 2 (VEGFR2) and annexin-A4 (ANX-A4), were reduced (27). VEGFR2 and ANX-A4 both enhance proliferation, migration, and survival of the endothelial cells critical for placental blood vessels, suggesting that maternal and/or fetal blood flow that underlies many other placental functions may be dysregulated A trend increase of the pro-inflammatory cytokine, TNFα, in placenta after alcohol exposure also suggests that placental processes sensitive to inflammation, such as serotonin production, may be affected. This study reveals different aspects of placental abnormality than other assessments showing a higher rate of placenta accreta with gestational alcohol exposure (28). Placenta accreta occurs when trophoblast cells invade the uterine wall abnormally, which suggests altered initial placentation due to alcohol exposure. Placenta accreta can lead to complications during delivery and may be managed by pre-term cesarean delivery, both of which are linked to increased neurodevelopmental risk for children (29).

The aforementioned studies demonstrate examples of common maternal conditions with placental abnormalities that have also been linked to neurodevelopmental abnormalities of offspring. There are many other factors that can influence placental changes and therefore development of offspring; for example, regardless of maternal physiology, infant neurodevelopment has also been correlated with placental epigenetic variation (30). What is apparent from these studies is that the placenta's role is more than a waystation for the fetal brain to be exposed to molecules from maternal circulation. The impact of placenta nutrient transport, serotonin production, and hormone regulation are significant, as general development of the fetus has been found to be negatively influenced when these functions are abnormal (31). Additionally, a range of maternal physiological stresses impact placenta function (gestational diabetes mellitus, maternal infection). These forms of maternal stress and others may have overlapping neurodevelopmental impacts on offspring because of overlapping placental abnormalities.

## Linking Placenta and Neurodevelopment

In the examples discussed above, maternal conditions during pregnancy are linked both with improper function of the placenta as well as with increased likelihood of neurodevelopmental problems in offspring. The critical nature of the placenta for neurodevelopmental changes is hypothesized from such descriptive studies, but few studies have been able to causally connect placental abnormalities directly to altered brain development.

The impact of alcohol on placental growth factor (PLGF) has been explored for its mechanistic role in disruptions of fetal brain vasculature in mice. With CRISPR-Cas9 mediated over-expression of the PLGF gene in placenta, the reduced proportion of cerebrocortical radial vessels with maternal alcohol consumption is corrected to normal levels (32).

Maternal immune activation (MIA), a risk factor for ASD, involves elevation of maternal IL-6, a proinflammatory cytokine responsive to infections. Sophisticated work has shown that the impact of IL-6 on the placenta is the critical mediator of MIA effects on offspring neurodevelopment (20). A specific transgenic removal of placental IL-6 receptor, through the CYP19Cre driver (a transgene under the control of the aromatase cytochrome P450 19), resulted in offspring protected from the impacts of MIA in brain and behavior. Other findings from this work suggest that IL-6 signaling may play this critical role because it impacts placental angiogenesis and vascular permeability, hormone production, or further inflammatory signaling cascades in fetal circulation.

Another study has implicated the placenta's adaptation to nutrient deprivation (i.e., maternal starvation) as a way to protect offspring hypothalamus neurodevelopment—a critical brain region for hormonal regulation. During mouse gestation, expression of the gene *Peg3* is coordinated in the fetal hypothalamus and placenta, coordinating multiple other genes that regulate both placental and hypothalamic development (33). Following food deprivation, this pattern is uncoupled, with *Peg3* expression increased in fetal hypothalamus and decreased in placenta. This study demonstrates also that this maternal starvation stress advances hypothalamic cell growth, despite the reduction in nutrients due to breakdown of placental cells from decreased *Peg3* expression. Poor neurodevelopmental outcomes in the setting of maternal starvation may represent failure of this protective mechanism.

Maternal inflammation can be linked through the placenta to disrupted serotonin development in the fetal brain, particularly in the forebrain where serotonin plays a role in emotional regulation circuits. Specifically, MIA disrupts placental tryptophan metabolism, including altered expression of genes that synthesize serotonin [tryptophan hydroxylase 1 (TPH1) and indoleamine 2,3-dioxygenase 1]. Interestingly, controlled investigations of isolated placental and brain tissue after MIA also shows that TPH1 activity is increased only in placenta, but serotonin levels increase in fetal brain. Ultimately this increase in serotonin delivered to fetal brain suppresses normal outgrowth of fetal serotonin axons (34).

## Conclusion

The placenta has a temporary role in development during gestation—despite its time-limited presence, impacts of its function are clear well into adulthood. The impact of maternal illness or exposures during pregnancy on neuropsychiatric functioning of the next generation may well be heavily influenced by the health and performance of the placenta. If prenatal brain developmental changes from maternal physiological stresses that contribute to ASD, cognitive delays, or other neuropsychiatric problems originate in the placenta, finding ways to protect its structure and function are critical. Moreover, the placenta is a much more accessible biological target than the developing fetal brain for such interventions. Therefore, understanding mechanisms by which it influences the fetal brain, such as inflammatory signaling and serotonin production, will allow for more protective measures to be developed for healthy brain development in children.

## Author Contributions

SK and HS conceived the manuscript. SK, HS, and SM wrote the manuscript. All authors contributed to the article and approved the submitted version.

## Conflict of Interest

The authors declare that the research was conducted in the absence of any commercial or financial relationships that could be construed as a potential conflict of interest.
